# Impact of a health educational interventional program on reducing the head lice infestation among pupils in an elementary school of a sub-tropical region: a quasi-experimental study

**DOI:** 10.1186/s12887-022-03492-y

**Published:** 2022-07-18

**Authors:** Mohsen Najjari, Mohammad Amin Gorouhi, Hossein Zarrinfar, Bibi Razieh Hosseini Farash, Jamshid Jamali, Elham Moghaddas, Mohammad Ebrahimipuor

**Affiliations:** 1 Department of Parasitology and Mycology, Faculty of Medicine, Mashhad University of Medical Sciences, Mashhad, Iran; 2 Research Center Of Tropical and Infectious Diseases, Kerman University of Medical Sciences, Kerman, Iran; 3 Allergy Research Center, Mashhad University of Medical Sciences, Mashhad, Iran; 4 Department of Biostatistics and Epidemiology, School of Health, Mashhad University of Medical Sciences, Mashhad, Iran; 5 Research Center for Hydatid Disease in Iran, Kerman University of Medical Sciences, Kerman, Iran

**Keywords:** Pediculosis, Training programs, Primary schools, Infestation, Iran

## Abstract

**Background:**

Pediculosis is an important social challenge that can be caused by human head louse, *Pediculus humanus capitis.* This infestation is cosmopolitan, especially in countries with low hygiene and sanitation. Regular health education classes for students and their parents are required to reduce this infestation in schools and families. This study aimed to evaluate the impact of a health education intervention program on reducing head lice among pupils of an elementary school.

**Methods:**

In a quasi-experimental study, a total of 880 elementary school girls were screened for pediculosis in (2017–2018). The prevalence of pediculosis follow-up continued monthly until the end of the school year after a two months educational intervention course. Visual inspection was applied for initial diagnosis of infection. The suspected cases were confirmed by wood lamp and potassium hydroxide (KOH) microscopic examination. Also eight questions about diagnostic, epidemiology, treatment and prevention were answered in a questionnaire by 50 volunteer parents before and after the training intervention course, to evaluate their general knowledge and measure the effectiveness of learning. All data were statistically analyzed using SPSS software (version 20.0, SPSS, Inc., Chicago, IL, USA). McNemar’s test was also applied to investigate the prevalence rate before and after the intervention. A *p* -value of less than 0.05 was considered as statistically significant.

**Results:**

At the beginning of study, pre-intervention prevalence of pediculosis among pupils was 8.4% (49/594). The mean age in all pupils was 9.86 ± 1.83 years old and the most infestation was shown in fourth-grade students with 10 years old. Analysis of statistics demonstrated a significant difference between having infestation and the number of members in the families. Post-intervention phase led to a decreased prevalence of pediculosis in pupils to 3% (8/594) (*p*-value < 0.05). Based on statistical analysis of questions before and after the training in the questionnaire, a significant increase of parental knowledge was observed on the prevention program of pediculosis (*p*-value < 0.001).

**Conclusion:**

The prevalence of pediculosis was significantly reduced following the educational interventions in the school. The applied interventions may be implemented in other residual centers to get rid of this important infestation.

## Background

Head lice infestation is an important social challenge, which can be developed by *Pediculus humanus capitis* as a human head louse. This infestation is cosmopolitan, especially in countries with low hygiene and sanitation [1]. These obligate ectoparasites of humans have been well-known in antiquity and are associated with some life-threatening diseases including epidemic typhus, relapsing fever, and trench fever [2]. As a rapid spreading infestation, pediculosis is considered the most common contagious disease among school children afterward common cold virus [3]. Spreading of lice is more commonly due to direct contact with the hair of infested people. However, other uncommon routes such as contact with clothing and inanimate objects and personal belongings of infested persons are reported throughout the world [4]. Living in more crowded places including schools, orphanages, and other residual centers increase the risk of infestation. Subsequently, infested people in the mentioned centers spread lice between their families as well as societies [5]. However, parents’ education level, family financial status and geographical location of the schools have been found to have no association with the spreading of head lice [6]. On the other hand, head lice infestation has been more reported in females than males [7]. Untreated pediculosis can result in itching, hives, repeated wounding of head and body skin, mental health (nightmares), and allergy in infected individuals [3, 8]. These mentioned symptoms are more common in chronically infected patients, and it is due to toxicity of the insect saliva [9]. Head lice outbreaks and its obvious symptoms are more in children and pre-school pupils and lead to a negative attitude by their classmates and friends [10]. Due to the potential spreading of pediculosis from infested people to society and their families, treatment and follow up of head lice infestations should be more emphasized in each region [11]. For some people, the pediculosis management and treatment is expensive and laborious. Thus, it should be considered as a community-based approach to cover families, schools, health care professionals and the governments [12, 13].

Previous studies in Iran demonstrated a considerable prevalence of head lice infestation in all provinces or geographic regions [14–21]. This infestation has increased significantly over time in both girls and boys, and it has varied from 1.72% in 2015 to 3.42% in 2018 [22, 23]. Effective communication between parents and school health instructors about head lice can have an essential role in the control of pediculosis. Regular health education classes for students and their parents are required to reduce this infestation in schools and families [24, 25]. Despite the efforts of health authorities in Iran, the incidence of head lice infestation is increased in school children under the age of fifteen, particularly in female elementary school pupils [20, 23]. Moreover, the incidence of pediculosis is increased in Mashhad metropolis, the capital of Khorasan-Razavi Province. Due to being a hospitable city and a destination for religious tourism, this city has been a major endemic focus of pediculosis in Iran [26].

This study aimed to evaluate the effectiveness of parental and pupils’ knowledge and attitude on reducing head lice among pupils in elementary schools in northeastern Iran. For this goal, a training-oriented process has been designed for increasing the awareness of teachers, pupils, and parents to prevention and control approaches of pediculosis in Mashhad city, northeastern Iran.

## Materials and methods

### Study area

Mashhad is a city in northeast Iran, known as a place of religious pilgrimage and one of the metropolises cities of Iran. This cosmopolitan city Located in 36.2605° N, 59.6168° E. It is, the capital of Khorasan-Razavi Province.

### Methods

In this quasi-experimental study, in order to evaluate the effect of parental and student education on reducing pediculosis, two primary schools (*n* = 880) was randomly selected from a socio-economic medium urban area of Mashhad city as intervention and control groups by cluster and randomize sampling in (2017–2018). To estimate prevalence pediculosis in pupil, the sample size was determined according to the expected prevalence of pediculosis (12.5%) [27], which also already was obtained in our pilot study and using Cochran’s formula (prevalence rate), considering error of 5%, test power of 80% and relative error of p/4, the minimum predictable sample size was determined about 880 cases. About one third was considered to the control group (*n* = 311), and two thirds (*n* = 594) to the intervention group. The prevalence of pediculosis estimated before and after two months in an interventional education program for students A developed form, demographic data, weight, height, hair length, body mass index (BMI), parents’ education and occupations, and the number of family members were recorded. A monthly one-hour meeting maintenance program in order to follow pediculosis was considered in which parents and students took part in each class and educational items were reviewed in the presence of the health liaison. Successful and unsuccessful experiences were shared, thus monitoring of the contamination and its frequency continuously was observed.

### Sampling and detection

The visual inspection was applied for initial diagnosis of pediculosis infection by the school’s competent public health consultant. Followed by, the suspected cases were confirmed by wood lamp and direct microscopic examination. The wood lamp examination determined the existence of lice eggs (nits) in greenish-yellow fluorescents that attached to the base of the infested hair shaft. During the direct microscopy examination, hair samples were trimmed with scissors, and were clarified with a drop of 10% potassium hydroxide (KOH) on a microscopic slide, covered with a sterile coverslip (with a maximum magnification of 100 × and 400 ×).

All elementary school students who are periodically (monthly) visited by the hair regarded as inclusion criteria and unwillingness to participate in the program and not consuming permethrin shampoo in the month before sampling were considered as sampling exclusion criteria in the study. Detected infestations in the pupils were categorized into past, recent and present infestation cases. Detecting dead eggs and/or nits more and less than 1 cm from the scalp is categorized in past and recent infestation, respectively. The existence of lice and/or eggs was considered as a present infestation. The light infestation was considered in cases with less than 5 lice and a few eggs. Numerous lice and eggs was considered as heavy infestation, and the existence of lice, eggs and numerous lice eggs and nits was detected as heavy long-standing infestation [28]. In order to reduce the effect of confounding factors in the study, an elementary school in the same area and similar socio-economic level was selected as the control group in which no educational intervention was performed. The prevalence of contamination by the study method was measured in the control group.

### Interventions

In two steps, the interventions were applied in eradication of infestation due to case detection, and training-oriented prevention program. These implementation steps were applied with considering ethical standards about infested cases and their families. The detected cases were referred to health centers for completing the treatment courses. Simultaneously, the transmission, prevention, and control of these ectoparasites were completely explained to their parents and students in a two months program by specialists as well.

### Students’ training program

The main students’ training program took place in 4 sessions in two consecutive months. This training program was conducted during 35 to 45 min training sessions in the form of lectures, PowerPoint presentations, group discussions, questions and answers, and the distribution of educational pamphlets. The first session was dedicated to present the initial introduction and morphology of the human lice through a PowerPoint file and students’ drawing of it. The second session was dedicated to introducing the life cycle and how to feed, as well as the signs and symptoms of head lice infection through a lecture and PowerPoint presentation. The lessons of the previous session were reviewed by the students. The questions and answers were performed. The third session was devoted to lectures and PowerPoint presentations on how to treat with permethrin shampoo and the introduction of a doctor, clinic and laboratory at the place of residence and how to examine and sample head lice. The lessons of the previous session were reviewed by the students. The questions and answers were answered. The fourth Session dedicated to the prevention of infestation due to regarding personal hygiene including daily hair combing using personal brush, ironing their scarves, veils and cloths covering the coat and jacket in nylon bags prior to placing in classroom hanger. and how to regard personal hygiene in other public places such as subways, mosques, swimming pools, hotels and public accommodation centers and even the homes of relatives. Moreover, the holding justification one hour meetings were done for school managers and teachers to provide solutions and recommendations to manage this infestation monthly. The educational content of these part-time sessions were focused more particularly for classes with a high load of head lice infestation.

### Parents’ training program

Fifty parents who participated in the training sessions voluntarily, participated in the survey and asked eight questions in a standard questionnaire, whose validity and reliability had already been confirmed by the health belief model (HBM) [29]. The subjects were the aspects of recognizing a developed questionnaire (according to Dehghani et al. study, 2018) [29], with eight questions about diagnostic, epidemiology, treatment and preventions aspects were filled by parents before and after training, to evaluate their general knowledge and measure the effectiveness of learning.

The parents’ training program took place in 4 sessions in parallel to the pupil training program in two consecutive months. This training program was conducted during 45 to 60 min training sessions in the form of lectures, PowerPoint presentations, questions and answers and group discussions. The first session was dedicated to present the initial introduction and morphology and epidemiology of the human lice in Iran and abroad through a PowerPoint file. The second session was dedicated to introducing the life cycle, feeding, reproduction as well as the signs and symptoms of head lice infestation through a lecture and PowerPoint presentation. The questions and answers were performed. The third session was devoted to lectures and PowerPoint presentations on pathogenicity and diseases transmitted by lice and the introduction of the doctors, clinic and laboratory related to the project and the role of each and questions were answered. The fourth Session dedicated to the prevention measured and regarding personal hygiene including, The need for bathing twice a week and observance of hygienic aspects and monitoring of children’s hair in terms of lice eggs, daily hair combing using personal brush, ironing their scarves, veils and cloths and how to regard family hygiene in other public places such as subways, mosques, swimming pools, hotels and public accommodation centers and even the homes of relatives were discussed.

### Statistical analysis

All data were statistically analyzed using SPSS software (version 20.0, SPSS, Inc., Chicago, IL, USA). Relative frequencies were determined and data were expressed as mean ± standard deviation (SD). Shapiro–Wilk test was used to check the normality of quantitative variables. Mann Whitney U test was used to test the null hypothesis, and the Chi-square test was used to compare the relations and means. McNemar’s test was also applied to investigate the prevalence rate before and after the intervention. A *p* -value of less than 0.05 was considered as statistically significant.

## Results

### Before interventions

**Fig. 1:**
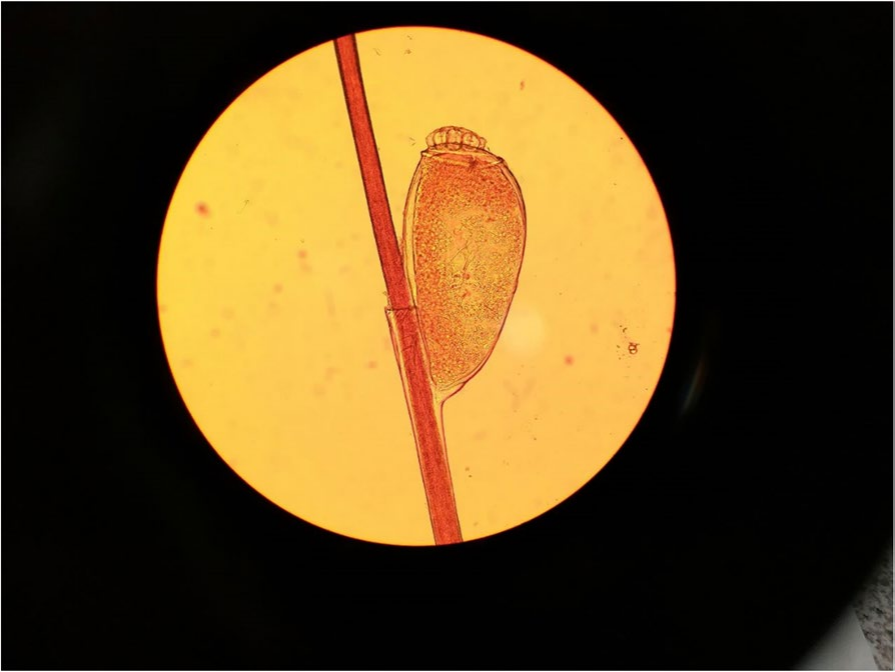
Laboratory identification of nit on the base of hair strand (KOH 10%), followed by direct microscopic examination (400 × magnification)

At the beginning of study in autumn, the overall prevalence of pediculosis among the pupils was 8.4% (49/594). The mean age in all pupils was 9.86 ± 1.83 years old and the most infestation was shown in fourth-grade students with 10 years old (Table 1). The descriptive statistics for age, height, weight, body mass index (BMI), number of family members, and frequency of infestation in before and after course are shown in Table 2. There was no statistically significant difference between having head lice and age, weight, height, and BMI of pupils. The education level and occupation of the pupil’s parents are characterized in Table 3. The number of family member’s average among studied pupils was 4.30 ± 0.93, and the infestation frequency’s average in four examinations was 1.08 ± 0.84. The analysis of statistics demonstrated a significant difference between having infestation and the number of family members. Moreover, the families with larger populations had more head lice infestation. The findings demonstrated a more infestation in long haired (62%) pupils, but without any statistically significant difference. The severity of infestation in 55% of the infested cases was light with a low number of 1–5 nits. Heavy infestations were observed in almost all grades. Figure 1 shows an instance of the presence of a nit attached to the hair shaft of an infected pupil in a direct microscopic examination (wet mount).

**Table 1:** Infected and non-infected cases among study group according to their different educational grade

School grade (age)	Negative cases	Positive cases Less than 5 Lice (Low)	Positive cases Between 5 and 10 nit and lice (Moderate)	Positive cases More than 10 lice (High)	Total
Pre-school (6)	59 (93.65%)	2 (3.17%)	0 (0.00%)	2 (3.17%)	63
Grade 1 (7)	85 (92.39%)	4 (4.35%)	1 (1.09%)	2 (2.17%)	92
Grade 2 (8)	90 (91.84%)	4 (4.08%)	2 (2.04%)	2 (2.04%)	98
Grade 3 (9)	101 (90.18%)	5 (4.46%)	4 (3.57%)	2 (1.79%)	112
Grade 4 (10)	103 (89.57%)	8 (6.96%)	3 (2.61%)	1 (0.87%)	115
Grade 5 (11)	107 (93.86%)	4 (3.51%)	1 (0.88%)	2 (1.75%)	114
Total	545 (91.75%)	27 (4.55%)	11 (1.85%)	11 (1.85%)	594

**Table 2:** Different characteristics of pupils, before interventions for head lice infestation

Characteristic	Non-infected (Mean ± SD)	Infected (Mean ± SD)	Total (Mean ± SD)	*P*-value
Age	9.68 ± 1.94	10.4 ± 1.7	9.86 ± 1.93	0.44
Weight	36.07 ± 12.51	34.24 ± 12.86	35.17 ± 12.65	0.44
Height	138.97 ± 12.91	137.02 ± 13.87	138.02 ± 13.36	0.55
BMI	18.26 ± 4.27	17.65 ± 3.78	17.96 ± 4.03	0.51
Number of family members	4.06 ± 0.78	4.56 ± 1.01	4.30 ± 0.93	0.003
Frequency of infestation in four time hair examination	0.00 ± 0.00	1.72 ± 0.93	1.08 ± 0.84	< 0.001

**Table 3:** Occupation and education level of parents of studied pupils for head lice infestation

		**Non-infested (%)**	**Infested (%)**	**Total (%)**	***P*-value**
**Father’s education**	Less than high school diploma	26 (50%)	15 (30%)	41 (40.2%)	0.04
	High school diploma	19 (36.5%)	19 (38%)	38 (37.3%)	
	University degree	7 (13.5%)	16 (32%)	23 (22.5%)	
	Total	52	50	102	
**Mother’s education**	Less than high school diploma	17 (32.7%)	12 (24%)	29 (28.4%)	0.5
	High school diploma	28 (53.8%)	28 (56%)	56 (54.9%)	
	University degree	7 (13.5%)	10 (20%)	17 (16.75)	
	Total	52	50	102	
**Father’s occupation**	Self-employed	40 (76.9%)	37 (74%)	77 (75.5%)	0.93
	Retired/Unemployed	3 (5.8%)	3 (6%)	6 (5.9%)	
	Employee	9 (17.3%)	10 (20%)	19 (18.6)	
	Total	52	50	102	
**Mother’s occupation**	Self-employed	1 (1.9%)	4 (3.08%)	5 (4.9%)	0.44
	Housewife	46 (88.5%)	41 (82%)	87 (85.3%)	
	Employee	5 (9.6%)	5 (10%)	10 (9.8%)	
	Total	52	50	102

### After interventions

The interventions lead to decreasing prevalence of pediculosis in pupils from 8.4% (49/594) in the first of fall and winter to 3% (8/594) by the end of spring. This decrease occurred with a statistically significant difference (*p*-value < 0.05). Moreover, according to the data extracted from the questionnaires, 97% of the parents believed that the training courses were useful and effective in the raising of awareness about pediculosis. The prevalence of pediculosis in control school showed the rates of infestation 9%, 12%, and 11% in autumn, winter, and spring, respectively. Education was able to elevate the attitude of parents on 8 different designed questions in the field of diagnosis, epidemiology, treatment and prevention of pediculosis significantly. Table 4 shows the results of interventions about head lice infestation among the parents of pupils.

**Table 4:** The results of interventions about head lice infestation among the parents of pupils, before and after interventions

**Variables** **(questions)**	**Scope**	**Before** **Intervention**	**After** **Intervention**			***P*-value**
			**No**	**Yes**	**Total**	
Checking your child hair for infestation in home	**Diagnosis**	No	6 (12%)	26 (52%)	32 (64%)	<0.001
		Yes	2 (4%)	16 (32%)	18 (36%)	
		Total	8 (16%)	42 (84%)	50	
Understand transition routes of pediculosis	**Epidemiology**	No	3 (6%)	40 (80%)	43 (86%)	<0.001
		Yes	2 (4%)	5 (10%)	7 (14%)	
		Total	5 (10%)	45 (90%)	50	
Using personal accessory	**Prevention**	No	6 (12%)	38 (76%)	44 (88%)	<0.001
		Yes	4 (8%)	2 (4%)	6 (12%)	
		Total	10 (20%)	40 (80%)	50	
Ironing yours and your child's clothes regularly	**Prevention**	No	6 (12%)	26 (52%)	32 (64%)	<0.001
		Yes	4 (8%)	14 (28%)	18 (36%)	
		Total	10 (20%)	40 (80%)	50	
Understand treatment of pediculosis	**Treatment**	No	1 (2%)	26 (52%)	27 (54%)	<0.001
		Yes	2 (4%)	21 (42%)	23 (46%)	
		Total	3 (6%)	47 (94%)	50	
Participate in educational programs at school about pediculosis prevention	**Prevention**	No	2 (4%)	45 (90%)	47 (94%)	<0.001
		Yes	1 (2%)	2 (4%)	3 (6%)	
		Total	3 (6%)	47 (94%)	50	
Use the bath weekly	**Prevention**	No	3 (6%)	30 (60%)	33 (66%)	<0.001
		Yes	2 (4%)	15 (30%)	17 (34%)	
		Total	5 (10%)	45 (90%)	50	
Use the common blanket and cloth	**Prevention**	No	3 (6%)	20 (40%)	23 (46%)	0.036
		Yes	8 (16%)	19 (38%)	27 (54%)	
		Total	11 (22%)	39 (78%)	50	

## Discussion

Despite global improvements in the hygiene and sanitation of societies, pediculosis is reported from both developed and developing countries. This is considered as a growing neglected infestation in many regions of the world, especially developing countries. Pediculosis can occur in schools and other residual centers of rural and urban areas (5).The high prevalence (8.4%) of pediculosis in recent studies is in line with the previous studies in Iran, which demonstrated prevalence from 1.6% to 67% in different provinces [17, 19, 27]. No statistically significant difference between pediculosis and age, weight, height, and BMI of pupils. There was also no relationship between the variables in previous studies in Iran and other countries [22, 30]. However, this study showed that all pupils in schools are at risk of head lice infestation. On the other hand, it was shown that the number of individuals in the families is an effective factor on more infestation. This finding agrees with the previous study in Iran with an association between mentioned variables [27]. In line with other studies in Iran, despite more infestation in long hair pupils (62%), no statistical difference was indicated in the current study [27, 30]. Heavy and light pediculosis was demonstrated in all infested pupils in every age group. Although, the occupation and education level of parents have been shown to have no effect on the frequency of pediculosis [20]. According to the study results, it seems any increase in the education level of the family members can lead to serious changes in the attitude, viewpoint and behavior of all family members about pediculosis. However, its reasons have not been explained exactly, and need additional clarification on the specific reasons in various studies. High prevalence of pediculosis in Mashhad elementary schools could be a reflection of the family’s lifestyle changes in recent years. This problem might be related to increasing use of music player devices and using tools such as earphones and headphones, which are usually shared between family’s members. Moreover, other urban civilization phenomena such as the subway, which has been more popular, can be effective as a crowded and closed place in the formation of uncontrolled outbreaks. Furthermore, the use of schools to welcome local rural pilgrims in recent years on various occasions has led to temporarily accommodating large human populations in the metropolis of Mashhad. This event could be considered as a potential in creating an epidemic at the schools, particularly in winter that must be considered.

Interventions in the infested pupils and programming for the training of parents and pupils as well as teachers have a critical role in the control of pediculosis in each society [31, 32]. Improvement of parent’s awareness, knowledge, and perception about head lice, timely preventive health actions, and interruption of the parasite life cycle could lead to decrease of this infestation in the present study. For example, before intervention in our study, more than 86% of parents have no knowledge about transmission routes of pediculosis. While after the intervention, the ratio has changed significantly. Our findings are in line with studies of [29] and [33] that previously measured the level of parental learning in health education programs for pediculosis. Despite this significant decrease in the prevalence rate, the eradication was not achieved, which seems to be due to the short duration of intervention and control. Therefore, it is suggested to approve and implement some long-term 5-year plans for future projects. For example, Frankowski et al. could achieve a considerable success in this field, during a 6-year plan [11]. Furthermore, confounding factors in this study can be pointed to fungal diseases in such children age groups that mimic pediculosis. Although the prevalence of pediculosis in our study was the highest level of infection in the intervention group in autumn and winter, due to the intervention and its effects, the prevalence of pediculosis in the spring was lower in line of previous study Bauer et al. [34]. But it varies with the study of Kassiri et al. while in the control group the prevalence of pediculosis was constant in almost all seasons and has remained high [35].

Therefore, it should be distinguished specifically from superficial fungal diseases such as trichomycosis, tinea capitis, white piedra, black piedra and dandruff caused by *Malassezia* [36–38]*.* Interestingly in the current study we faced several misdiagnosed cases that later ruled out for pediculosis. Choosing the right treatment requires a prior accurate diagnosis (clinical and laboratory), because of the possibility of drug interactions and side effects. However, some physicians do empirical treatment regardless of patient referral to the laboratory for definitive diagnosis. In some cases, these infections may also be simultaneously [39]. Hence, in such cases it requires the collaboration of an entomologist/parasitologist with other specialists such as a mycologist and dermatologist.

And finally, some of the important results that we achieved in this study include: first, creating a sense of responsibility in pupils to convey and share useful information about prevention of infestation among their families, relatives and classmates. Second, the parent-teacher association (PTA) and its role to solve the school problems through scientific and research methods have been regarded. Third, use of the educational potentials of families, which led not to impose financial burden on families have been noticed, country health services, and school management. Fourth, to reduce of anxiety around the stressful situation for the parents and teachers, those were in daily contact with the infested pupils.

One of the notable points in this regard, there have not been ever conducted such studies on female elementary school students in this area. So, the findings of this study could increase our knowledge from the epidemiological aspects of disease, and could change the authorities’ attitude and focus from expensive case therapy toward cheap group training.

There are some limitations in our study that deserve mention: the collected data was a self-report by mothers and students files, thus recall bias is warranted. Also, we performed the studies in one urban area of Iran, thus our findings may have limited generalizability to other regions and cultures.

## Conclusion

This study provided evidence-based effective interventions for reducing head lice infestation in the elementary schools. Current study showed a successful practical approach, step by step with emphasis on process-oriented teaching to reduce pediculosis infestation using contribution of teachers, students and families as the core target groups for interventions. Fighting lice requires a comprehensive interaction and cooperation from home to school. Any medical and educational interventions or isolation and quarantine of children at home or school alone will not be effective in fighting head lice. We recommend such a developed prevention program. However, further studies are required to understand the effectiveness of these interventions in the control of pediculosis in other regions, due to the different geographical, cultural and climatic alterations.

## Data Availability

The datasets used and/or analyzed during the current study are available from the corresponding author on reasonable request.

## Abbreviations

KOH: Potassium hydroxide
HBM: The health belief model
SD: Standard deviation
BMI: Body mass index
PTA: Parent-teacher association
